# Lower vitamin D status may help explain why black women have a higher risk of invasive breast cancer than white women

**DOI:** 10.1186/s13058-020-01261-2

**Published:** 2020-02-21

**Authors:** William B. Grant

**Affiliations:** Sunlight, Nutrition, and Health Research Center, San Francisco, CA USA

To the Editor:

The recent article by Dania and colleagues reported that black women had a 33% increased risk for subsequent invasive breast cancer after diagnosis of lobular carcinoma in situ (LCIS) during 90 months of follow-up [1]. The authors suggested that further research might better understand the contributors to racial differences in developing invasive breast cancer following LCIS.

The authors did not consider the role of vitamin D in affecting risk of invasive breast cancer. Black women living in the USA have much lower 25-hydroxyvitamin D [25(OH)D] concentrations than do white women due to their darker skin [2].

There is evidence that breast cancer risk among black women is affected by 25(OH) D concentrations. For example, a prospective study from the Black Women’s Health Study found that the incidence rate ratio for invasive breast cancer for lowest vs. highest quartile of predicted 25(OH) D was 1.23 (95% CI, 1.04, 1.46) [3]. For another study, see Ref. 8 in [3]. More recently, Ref. 37 in [4] reported that in a clinical trial in which participants in the treatment arm were given 2000 IU/d vitamin D_3_, black participants had a 23% reduction [hazard ratio = 0.77 (95% CI, 0.59, 1.01)] in overall cancer incidence.

The evidence that vitamin D reduces risk of breast cancer was reviewed recently [4]. The strongest observational evidence comes from case-control studies. Breast cancer can progress rapidly to detectable size, and breast cancer is the only type of cancer with pronounced seasonal variation in diagnosis, with higher rates in spring and fall (Ref. 12 in [4]). One reason given for rejecting case-control studies as evidence for the effects of vitamin D on risk is that the existence of breast cancer, even if not diagnosed, may affect serum 25(OH) D concentration. Several studies are presented in [4] indicating that there is little evidence that disease status, including cancer and infectious diseases, significantly affects 25(OH) D concentration. However, the fact that the 25(OH) D concentration-breast cancer incidence relationships in 11 studies from seven countries have the same shape argues against that concern [5].

The proposed mechanisms whereby vitamin D metabolites reduce the risk of breast, colorectal, prostate, and overall cancer include surveillance of cells, reduced angiogenesis around tumors, and anti-metastasis actions (see references in [3, 4]). However, higher 25(OH) D concentrations have also been found associated with increased risk of colorectal and prostate cancer [4]. Further research is indicated to evaluate the UVB-vitamin D-cancer hypothesis.

## Data Availability

All data and material have been referenced and are readily available.
